# Evaluation of topical oclacitinib and nail trimming as a treatment for murine ulcerative dermatitis in laboratory mice

**DOI:** 10.1371/journal.pone.0276333

**Published:** 2022-10-18

**Authors:** Steven E. Davison, Kathryn M. Emmer, Beatrice Ugiliweneza, Leslie C. Sherwood

**Affiliations:** 1 Comparative Medicine Research Unit, School of Medicine, University of Louisville, Louisville, Kentucky, United States of America; 2 Department of Neurosurgery, School of Medicine, University of Louisville, Louisville, Kentucky, United States of America; 3 Kentucky Spinal Cord Injury Research Center, School of Medicine, University of Louisville, Louisville, Kentucky, United States of America; 4 Department of Health Management and Systems Science, School of Public Health and Information Science, University of Louisville, Louisville, Kentucky, United States of America; Freie Universität Berlin: Freie Universitat Berlin, GERMANY

## Abstract

Murine ulcerative dermatitis (UD) is a common, multifactorial skin disease of C57BL/6 and C57BL/6-background strains of mice. Many treatment options have been previously reported but have been variably successful and may interfere with specific research studies. Janus kinase (JAK) inhibitors, such as oclacitinib, have been used to treat allergic dermatitis in humans, dogs, and other species. Additionally, topical oclacitinib was shown to improve an induced model of dermatitis in mice. We hypothesized that topical application of oclacitinib in conjunction with hind limb nail trimming would improve UD lesion scores more than our institutional standard treatment regime using meloxicam, topical antibiotic ointment, and nail trimming or nail trimming alone. To test this, mice with naturally occurring UD were recruited to the study and assigned to one of three treatment groups (n = 14/group): nail trim only; nail trim plus meloxicam and topical triple antibiotic ointment; or nail trim plus topical oclacitinib. UD was assessed on days 1, 7, and 14 for all treatment groups, and scored based on a previously published scoring system that quantitatively scored UD lesions based on pruritus, character of the lesion, size of lesion, and location of lesion. Here we found that mean UD scores decreased from day 1 to day 7 and from day 1 to day 14 for all treatment groups. However, there was no significant difference in mean UD score between the treatment groups at any timepoint. These data show that topical oclacitinib and nail trimming together improved UD lesion scores comparably to our institutional standard treatment and nail trimming alone. However, further studies may be warranted to investigate other potential applications of oclacitinib to treat UD.

## Introduction

Murine ulcerative dermatitis (UD) is a common, multifactorial skin disease in C57BL/6 and C57BL/6-background strains of mice [1]. A clear etiology is not understood, but many contributing factors including age, sex, diet, and parasite infestation have been reported [1–12]. The high prevalence among C57BL/6 strains is suggestive of some level of genetic implication [1, 9]. Clinical signs of UD include epidermal scabs, crusts, and ulceration along with intense pruritus [10]. Self-trauma from pruritus perpetuates lesions and can result in secondary bacterial infections, ear pinnae amputation, and other sequelae such as infertility, weight loss, and reduced life span [10]. These lesions often lead to premature removal of mice from research studies or breeding colonies, and if not identified or left untreated, serves as a variable for researchers due to inflammation and infection.

Studies have evaluated various treatment options for UD including nail trimming, systemic medications, and/or topical therapies. Nail trimming alone as a conservative treatment can lead to resolution of some cases, with treatment success as high as 93% in one study [2, 3]. Topical agents such as 0.005% sodium hypochlorite, 1% silver sulfadiazine, and bacitracin-neomycin suflate-polymixin B sulfate ointment have had varied success [8, 9, 11]. The highest treatment success among these topical treatments was with 0.005% sodium hypochlorite, which resulted in a 71% cure rate when the solution was applied once daily [8]. Additionally, systemic non-steroidal anti-inflammatory drugs (NSAIDs) such as ibuprofen administered in drinking water and intraperitoneal maropitant citrate have both been shown to reduce pruritic behavior and improve healing of UD lesions [12, 13]. Furthermore, dietary supplementation such as vitamin E, marine red algae, and *N*-acetylcysteine may have both protective and negative effects on UD prevalence and severity [4, 6, 7, 9, 14]. However, nail trimming alone has had the highest reported treatment success [2].

Janus kinase (JAK) inhibitors are commonly used in veterinary medicine to treat allergic dermatitis in dogs by down-regulating pro-inflammatory and pruritogenic cytokines and controlling pruritus [15–17]. JAK inhibitors have also been used as therapeutic agents for pruritic inflammatory skin diseases in humans [18]. Oclacitinib is a nonselective JAK inhibitor with strongest inhibition against JAK1-dependent cytokines and shows minimal activity against JAK2-dependent cytokines in cellular assays, and is commercially available as the canine oral tablet, Apoquel^®^ [17]. In a pre-clinical study, both topical and oral administration of oclacitinib reduced pruritus in an induced mouse model of allergic dermatitis using BALB/c mice, and topical administration also reduced skin thickness and inflammatory response [19]. These results suggest there is therapeutic potential of using topical oclacitinib to treat spontaneous UD in laboratory mice.

We hypothesized that topical application of oclacitinib in conjunction with hind limb nail trimming will improve ulcerative dermatitis lesion scores more than our institutional standard treatment regime using meloxicam, topical antibiotic ointment, and nail trimming or nail trimming alone. If successful, topical oclacitinib could expand our treatment options for murine UD, thereby improving both animal welfare and research outcomes.

## Materials and methods

### Mice

All mice were housed at the University of Louisville on unrelated IACUC-approved protocols. Animals were recruited for this study between September 2020 and January 2021 once a veterinarian confirmed spontaneous skin lesions were consistent with UD and the animal was of C57BL/6 or C57BL/6-background. Animals with known experimental manipulations were not included in this study. Animals with unknown experimental histories were included if confirmed healthy by a veterinarian other than the skin lesions. Once confirmed, investigators voluntarily transferred the mice to this study where all activities were approved by the University of Louisville (UofL) Institutional Animal Care & Use Committee (IACUC) (Protocol number: 20766) with housing and care following *The Guide for the Care and Use of Laboratory Animals*, *8*^*th*^ *Edition* [20]. The UofL Animal Care and Use Program is accredited by AAALAC International. Mice with known birthdates ranged in age from 84 to 739 days old, 14 mice had unknown birthdates. Forty-five total C57BL/6 or C57BL/6-background strain mice were recruited to the study, including 11 males and 34 females.

Animals remained in their original housing room within 3 standardized vivaria to avoid transportation stress and were singly housed for treatment. All animals were housed on ventilated racks (Super Mouse 750^™^ Ventilated Racks & Cages, Lab Products, LLC, Seaford, DE) with corncob bedding (Bed-o cob, The Andersons, Maumee, OH) and ad libitum autoclaved rodent chow (LabDiet 5010, Lab Supply, Fort Worth, TX). Ad libitum reverse-osmosis water was either autoclaved and provided in bottles or supplied as hyperchlorinated water through an automatic-watering system (Systems Engineering, SE Lab Group, Inc., Napa, CA). Rooms were maintained at 22°C (±2°C) and 30–70% relative humidity, with a 12:12 h light:dark cycle.

Quarterly dirty-bedding sentinel and environmental testing of all vivaria were shown to be free of the following agents: mouse hepatitis virus, Sendai virus, minute virus of mice, murine parvovirus, reovirus type 3, epizootic disease of infant mice, pneumonia virus of mice, ectromelia virus, lymphocytic choriomeningitis virus, polyomavirus, mouse cytomegalovirus, mouse adenovirus, Theiler mouse encephalomyelitis virus, mouse kidney parvovirus, *Mycoplasma pulmonis*, *Filobacterium rodentium*, *Citrobacter rodentium*, *Salmonella* spp., *Clostridium piliforme*, *Pseudomonas aeruginosa*, *Klebsiella pneumonia*, *Klebsiella oxytoca*, endoparasites (*Syphacia* spp., *Aspicularis* spp.), and ectoparasites (*Myobia musculi*, *Myocoptes musculinus*, *Radfordia affinis*). Additional diagnostics for other potential dermal pathogens such as *Demodex* spp. was not performed.

A power analysis was performed ahead of animal recruitment with a goal to recruit 14 animals per group to have enough power to detect an effect size of 1.2 (very large). A total of 42 animals completed the study. One female mouse in the NMT group met humane endpoint at day 7, one female mouse in the NMT group was euthanized prior to completing treatment due to an unrelated medical problem, and one female in the NTO group was euthanized after completion of treatment but prior to the end of study due to a corneal ulcer. Animals who did not complete the study were removed from statistical analysis. No other animals died or were removed from study.

#### Treatment groups

**Fig 1:**
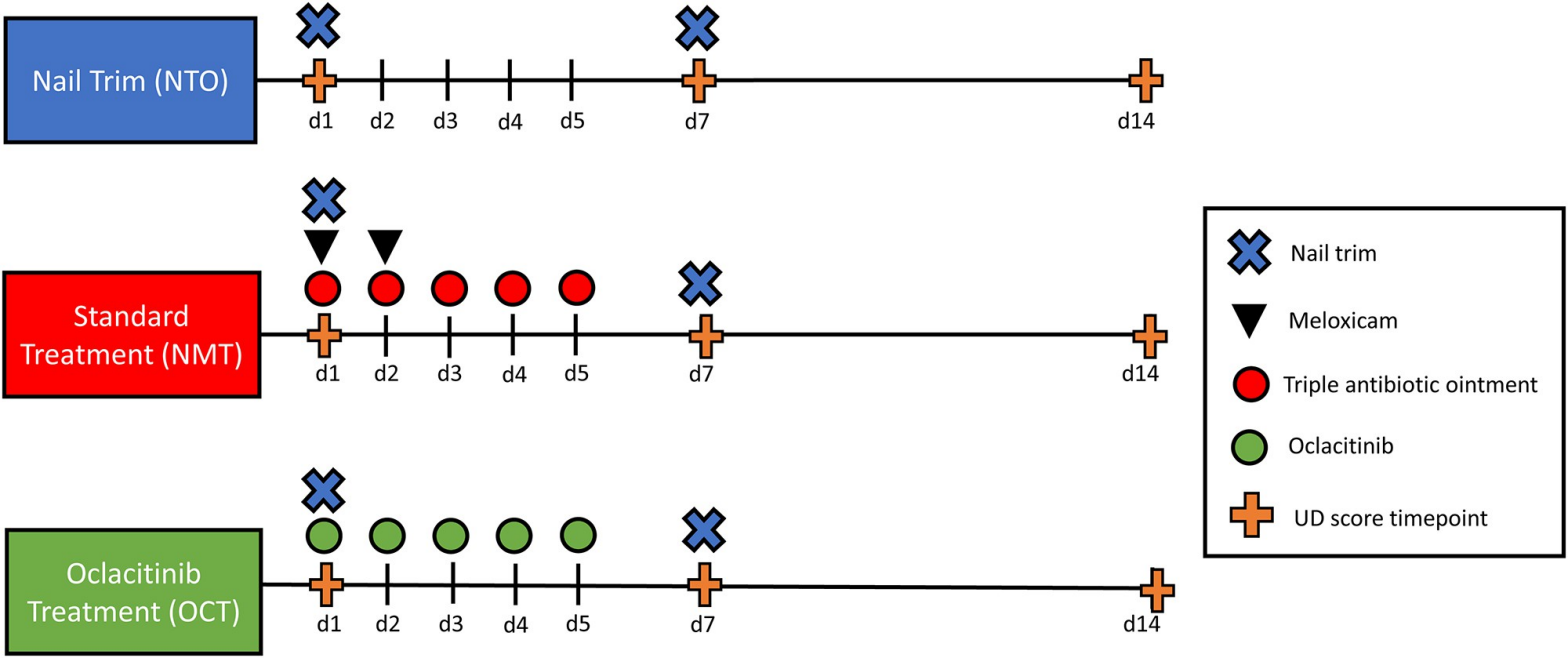
Treatment group and UD scoring timeline. Timeline for nail trims, medical treatments, and UD scoring for the 3 treatment groups: NTO (top), NMT (middle), and OCT (bottom).

**Fig 2:**
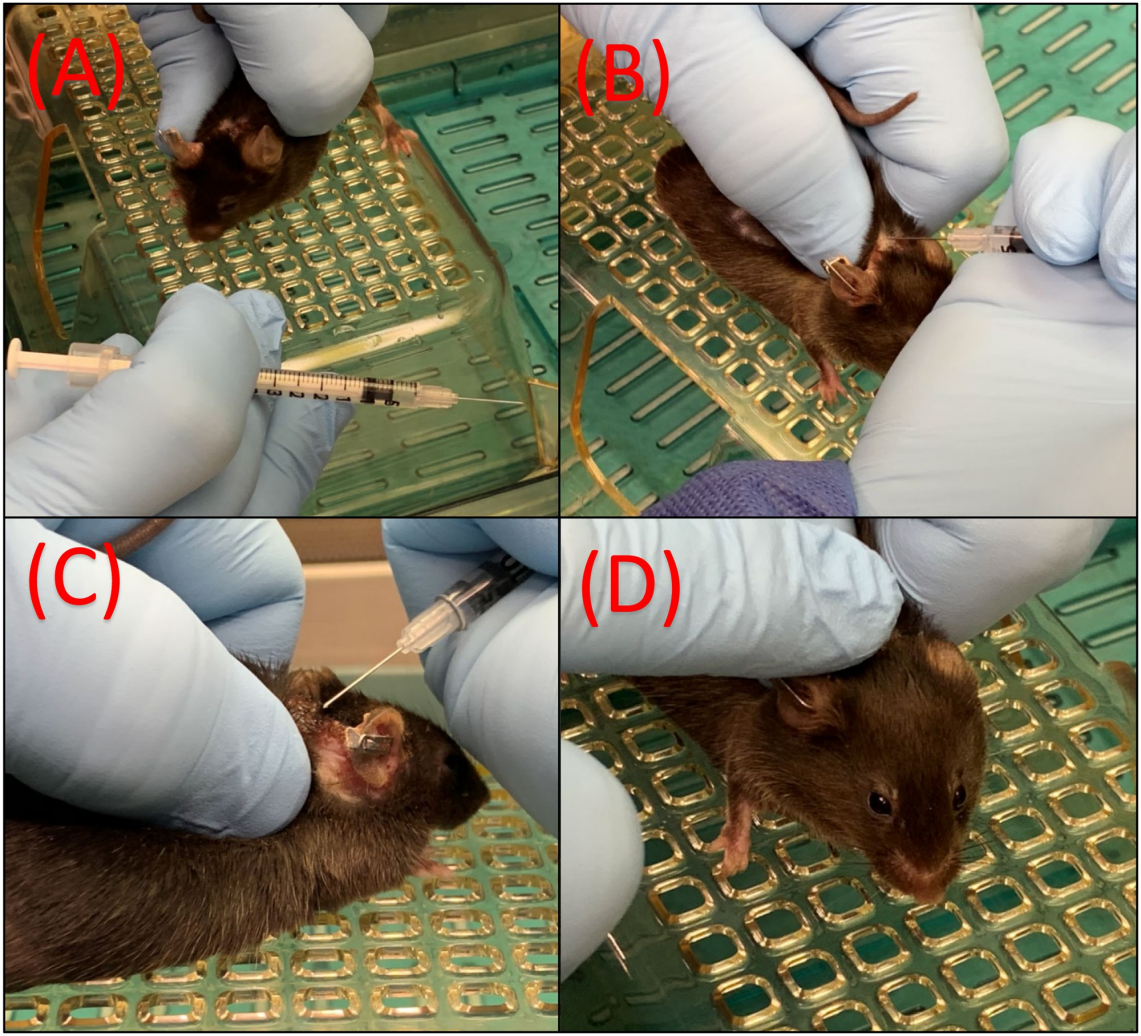
Topical administration of oclacitinib. (A) 50uL of 0.5% oclacitinib is draw up into an insulin syringe. The mouse is manually restrained, exposing any UD skin lesions. (B) The oclacitinib is dispensed onto the skin lesion. (C) Side profile showing application topically onto the skin lesion. (D) A gloved finger is used to disperse the liquid over all visible lesions.

Animals were assigned to one of three treatment groups (*n* = 14/group): nail trim only (NTO), nail trim plus meloxicam and topical triple antibiotic ointment (NMT), and nail trim plus topical oclacitinib (OCL) (Fig 1). Animals were not randomly assigned because an effort was made to evenly distribute males and females between groups. However, mice were reported and recruited to the study in an uncontrolled manor and assigned a treatment group before evaluating and scoring the animal. Mice in the NTO group received hind limb nail trimming as treatment for UD on days 1 and 7. The NMT group received hind limb nail trimming on days 1 and 7; 5mg/kg meloxicam (OstiLox^™^, Norbrook Group, Northern Ireland; diluted with sterile saline to 0.5mg/mL working solution) subcutaneously on days 1 and 2; and approximately 50uL of topical triple antibiotic ointment (CURAD Medline Industries, Northfield, IL; Bacitracin Zinc 400 units, Neomycin Sulfate 5mg, and Polymyxin B 5000 units per gram) on days 1, 2, 3, 4, and 5. Mice in the OCL group received hind limb nail trimming on days 1 and 7 and approximately 50uL of 0.5% topical oclacitinib (AdooQ^®^ Bioscience, Irvine, CA) diluted in DMSO (Valhoma Corporation, Tulsa, OK) similar to previous investigations on days 1, 2, 3, 4, and 5 [19]. Oclacitinib was drawn up and measured using an insulin syringe, then dispensed over the skin lesion(s) and rubbed in with a glove finger (Fig 2).

### UD scoring

**Fig 3:**
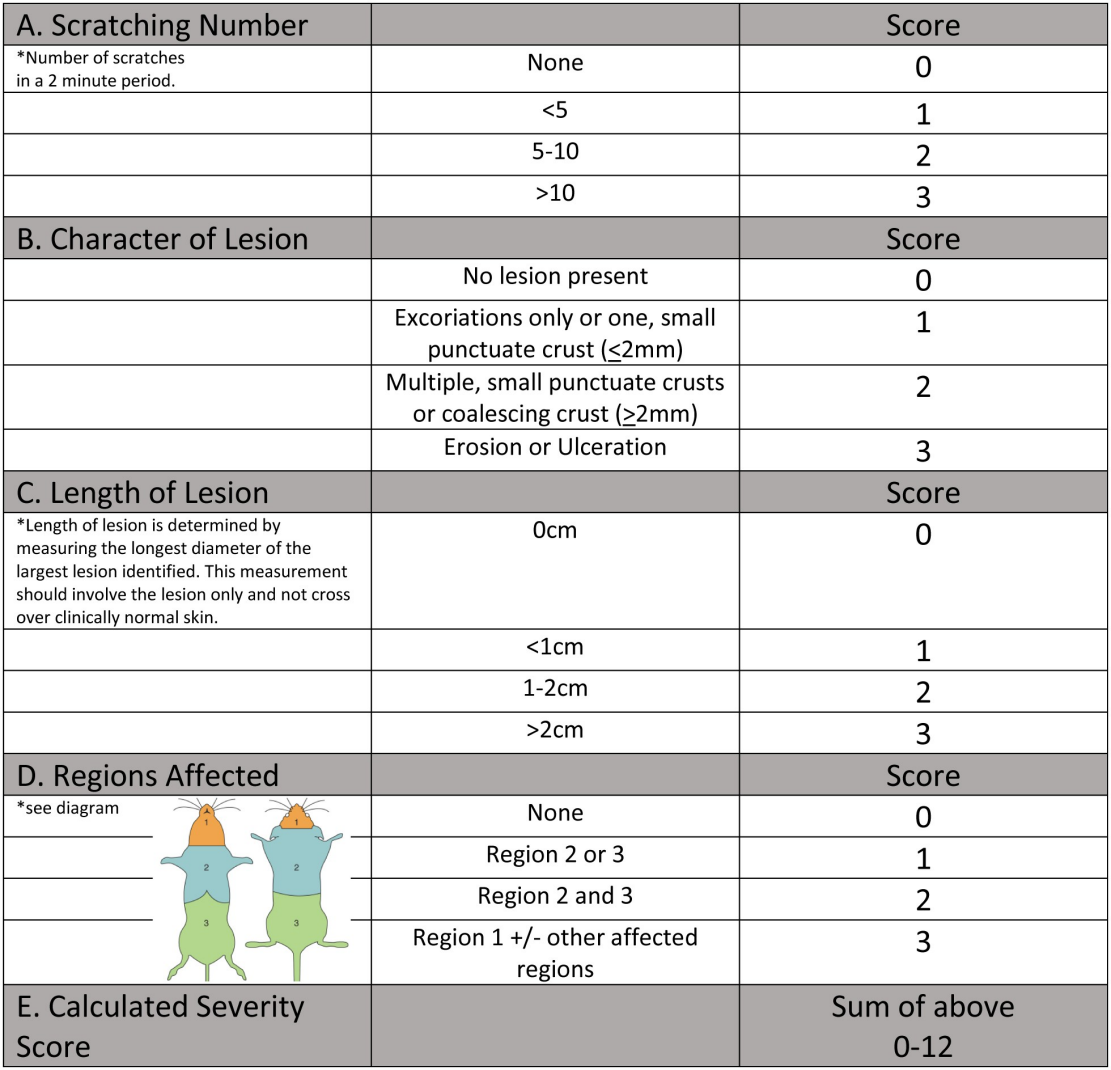
UD scoring system. Modified UD scoring system [5]. Image reproduced and modified from reference 5 with permission.

Using a modified scoring system previously reported (Fig 3), mice were scored on days 1, 7, and 14 [5]. Briefly, pruritus (measured as number of scratches in a 2-minute period), lesion character, lesion size, and lesion location were scored 0–3 and the totals were summated for an overall severity score (0–12). Mice receiving a score >9 were euthanized immediately due to meeting humane endpoint criteria. Treatment was otherwise not altered based on UD score. All scoring was performed by two veterinarians and one veterinary technician. Spot checks were randomly performed to ensure consistent scoring among individuals.

### Statistical analysis

The scores were analyzed with mixed linear models including a random intercept for each mouse to capture individual variability and random slope for measurement time (day 1, day 7, day 14) to capture individual change variability. The factors in the model were treatment (NTO, NMT, OCL), measurement time and their interaction. To obtain the changes within group over time, the differences across groups at each measurement time and the differences of changes across groups, linear contrasts were built on the interaction term. Estimates were presented with least square means along with their standard errors from the analytical model. Additionally, the three groups were compared in terms of the mice proportion that reached a score of 0 using the chi-square test and the time (in weeks) to get to that score using the log-rank test. All tests were 2-sided with a significance level of 0.05. Analyses were performed in SAS 9.4 (SAS Inc., Cary, NC).

## Results

### Sex and age of animals

Sex was not different between groups (*p* > 0.05). Age was different between the NTO and NMT groups (*p* = 0.0424), but the significance between mean score changes is unchanged when adjusted for age. Due to the fact that 14 animals had unknown ages, the mean UD scores reported below were not adjusted for age. Age-adjusted and unadjusted mean scores are reported in Tables 1 and 2.

**Table 1:** Age-adjusted mean UD scores and differences in mean UD scores.

Age-Adjusted Values				Changes					
	Day 0	Day 7	Day 14	Day 0–7		Day 7–14		Day 0–14	
	Mean ± SE	Mean ± SE	Mean ± SE	Mean ± SE	*p*-value	Mean ± SE	*p*-value	Mean ± SE	*p*-value
**NTO**	6.89 ± 1	2.75 ± 1	2.03 ± 1	-4.14 ± 1.37	<0.001	-0.71 ± 1.37	0.6031	-4.86 ± 1.37	<0.001
**NMT**	5.85 ± 0.75	1.93 ± 0.75	1.51 ± 0.75	-3.92 ± 1.04	<0.001	-0.42 ± 1.04	0.6911	-4.33 ± 1.04	<.0001
**OCL**	6.73 ± 0.86	3.96 ± 0.86	3.51 ± 0.86	-2.78 ± 1.21	<0.001	-0.44 ± 1.21	0.7136	-3.22 ± 1.21	<0.001

**Table 2:** Unadjusted mean UD scores and differences in mean UD scores.

Unadjusted Values				Changes					
	Day 0	Day 7	Day 14	Day 0–7		Day 7–14		Day 0–14	
	Mean ± SE	Mean ± SE	Mean ± SE	Mean ± SE	*p*-value	Mean ± SE	*p*-value	Mean ± SE	*p*-value
**NTO**	6.71 ± 0.69	2.93 ± 0.69	1.79 ± 0.69	-3.79 ± 0.98	<0.001	-1.14 ± 0.98	0.2453	-4.93 ± 0.98	<0.0001
**NMT**	5.71 ± 0.69	1.71 ± 0.69	1.36 ± 0.69	-4 ± 0.98	<0.001	-0.36 ± 0.98	0.7158	-4.36 ± 0.98	<0.0001
**OCL**	6.57 ± 0.69	3.07 ± 0.69	2.93 ± 0.69	-3.5 ± 0.98	<0.001	-0.14 ± 0.98	0.8842	-3.64 ± 0.98	<0.001

### Mean UD scores

All animals were scored upon recruitment to the study and then again at 7- and 14-days following initiation of the specified treatment regimen. At each timepoint, scores were analyzed within each treatment group and are reported as group mean ± standard error of the mean. The mean UD score for NTO was 6.71 ± 0.69 on day 1, 2.93 ± 0.69 on day 7, and 1.79 ± 0.69 on day 14; NMT was 5.71 ± 0.69 on day 1, 1.71 ± 0.69 on day 7, and 1.36 ± 0.69 on day 14; and OCL was 6.57 ± 0.69 on day 1, 3.07 ± 0.69 on day 7, and 2.93 ± 0.69 on day 14. There was no difference in mean UD score between the treatment groups at day 1, 7 or 14 (*p* > 0.05).

**Fig 4:**
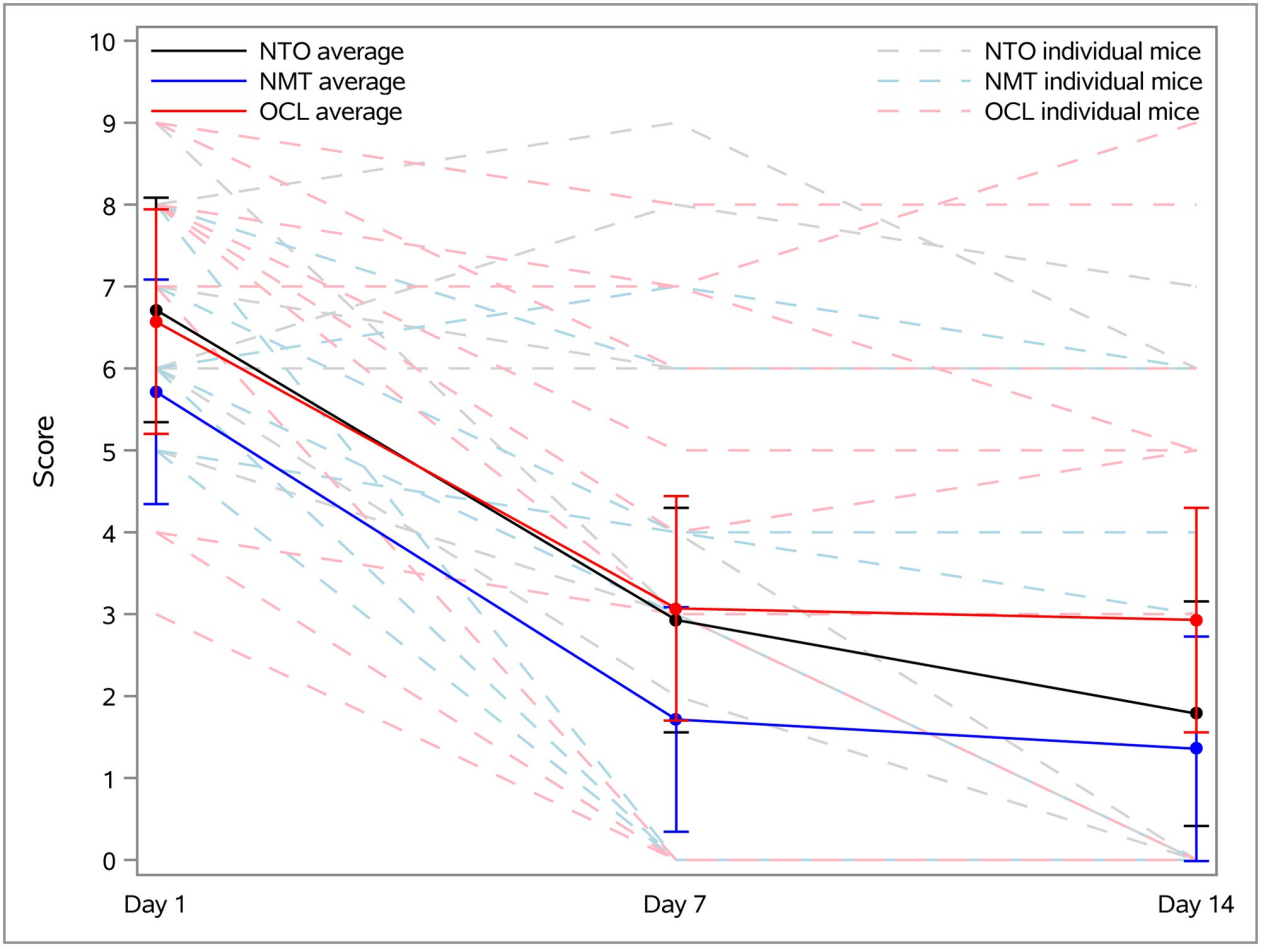
UD score change over time. UD score changes over study timepoints. The solid lines (NTO in black; NMT in blue; OCL in red) show the treatment mean scores while the broken lighter lines show individual animal scores. Data are presented as treatment mean ± standard error of the mean (SEM).

There was a significant decrease in mean UD score from day 1 to day 7 for the NTO, NMT, and OCL groups (-3.79 ± 0.98, *p* < 0.001; -4.00 ± 0.98, *p* < 0.001; -3.50 ± 0.98, *p* < 0.001 respectively). Similarly, there was also a significant decrease in mean UD score from day 1 to day 14 (-4.93 ± 0.98, *p* < 0.001; -4.36 ± 0.98, *p* < 0.001; -3.64 ± 0.98, *p* < 0.001 respectively). However, no significant decrease occurred between day 7 and day 14 for any treatment groups (*p* > 0.05). These results are shown in Fig 4.

### Time to resolution

We also evaluated the time required for an individual animal to achieve a UD score of 0, which equated to complete resolution of all skin lesions. By day 7, 43% of the NTO group (6/14), 64% of the NMT group (9/14), and 43% of the OCL group (6/14) reached of a score of 0. By day 14, 71% of the NTO group (10/14), 71% of the NMT group (10/14), and 50% of the OCL group (7/14) reached a score of 0. This proportion was not significantly different at either time point between treatment groups (*p* > 0.05).

## Discussion

Murine ulcerative dermatitis (UD) is frequently encountered in laboratory animal medicine given its prevalence in C57BL/6 & C57BL/6-background strains of mice [1]. Unfortunately, many of these cases are euthanized due to either treatment failure or interference with study goals. Recently, hind limb nail trimming has become a mainstay of UD treatment due to its efficacy and benign nature [2, 3]. Due to the importance of breaking the scratch-itch cycle and reducing inflammation, we were interested whether newer veterinary drugs such as JAK inhibitors would enhance UD treatment success in mice when used in conjunction with nail trimming. Oclacitinib is a popular JAK inhibitor administered in veterinary medicine and is sold under the trade name Apoquel^®^ [17]. Oclacitinib inhibits pathways involved in many pro-inflammatory, pro-allergic, and pruritic cytokines implicated in atopic dermatitis including interleukins (IL) 2, 4, 6, and 13 and is also involved in signaling IL-31, which has been shown to play a key role in canine pruritus [15]. In these ways, oclacitinib’s primary role is in reducing pruritus by these pathways but also has anti-inflammatory properties that are not yet well understood. Oclacitinib has previously been shown to reduce pruritus and inflammation when applied topically to dermatitis induced by toluene-2,4-diisocyanate in BALB/c mice [19]. However, it has not been investigated whether oclacitinib administration improves healing of spontaneous ulcerative skin lesions in laboratory mice.

In this study, we compared UD lesion healing when treated with nail trimming alone; with our current standard treatment of meloxicam, triple antibiotic ointment and nail trimming; or with topical oclacitinib in conjunction with nail trimming. Here we found that mean UD scores were significantly lower at day 7 and day 14 compared to day 1 for all treatment groups, demonstrating skin lesion improvement. However, all three treatments had comparable mean UD scores at the time points assessed (day 1, day 7, and day 14). This indicates that within our study parameters, adding topical oclacitinib or meloxicam and triple antibiotic ointment did not offer a significant benefit over nail trimming alone.

From a practical standpoint, oclacitinib administration was easy and simple. Subjectively, the authors did not notice a difference in grooming with the more liquid oclacitinib in DMSO solution as compared to the oil-based triple antibiotic ointment. The cost of oclacitinib treatment was more expensive than our standard treatment or nail-trimming alone. Oclacitinib cost approximately $3 per application, compared to less than $1 for the meloxicam and TAO used in the NMT group. There are opportunities to decrease the cost of the oclacitnib if other investigations explore utilizing commercial oral formulations rather than compounding a solution from powdered drug. The treatment cost of other novel UD treatments is not clear, however oclacitinib is likely as or more expensive than most other reported treatments [2–4, 6–8, 12, 13].

Nonetheless, this study served as a pilot to investigate the feasibility of using oclacitinib as a medical treatment in mice. Additional investigations exploring other administration routes, duration, and diluents may be warranted. In our study, the topical dose (0.5%) and diluent (DMSO) of oclacitinib was determined based partly on previous literature and also on product information (Adooq Bioscience, Irvine, CA). A recent study demonstrated that low dose DMSO (5mM) as a single treatment over 5 days accelerated skin wound closure, while higher concentrations (20mM) slowed wound healing in normal and diabetic mice [21]. The concentration of DMSO used as a diluent for oclacitinib in our current study and by Fukayuma et al. were both greater than the highest concentration used in that study [21]. There may be concern that DMSO can slow skin healing or cause irritation at a higher concentration or when applied more than once; however, only one animal in the OCL group had a score that subsequently worsened after the initial score. This was similar to the NMT group (one animal had a subsequent worsened score) and the NTO group (two animals had subsequent worsened scores). This suggests that there was no obvious worsening of the skin due to the DMSO administration under these study conditions. Nevertheless, the potential irritant effect of repeated DMSO administration may have masked some of the benefit of topical oclacitinib in terms of itching and inflammation. The current study did not include a DMSO control group, as the study by Fukuyama et al. found significant differences in cytokine production, scratching behavior, and ear swelling between JAK inhibitor topically treated (tofacitinib or oclacitinib) and control-treated animals [19]. Further studies may consider delivery of topical oclacitinib via other solvents including lower DMSO concentrations and compare results to DMSO alone.

As a naturally occurring disease and not experimentally induced, UD may respond to other treatment regimens outside the scope of this investigation. The treatment duration of 5 days described here was selected to mimic our standard treatment which typically includes 5 days of topical triple antibiotic ointment; however, clinical severity and treatment response may warrant a longer or shorter duration of oclacitinib application. We used this set schedule to reduce treatment variability in the study, but suspect that some of the more severe cases would have benefited from continuing topical oclacitinib on lesions past 5 days. Importantly, no gross adverse effects were observed following topical administration. Additionally, future investigations may consider monitoring animals for longer periods of time and more frequently to determine at what time point, if any, response plateaus.

One challenge for this study was capturing lesion severity and improvement given the inherent limitations of UD scoring. We adapted a scoring system published by Hampton et al., 2012, to objectively assess the animals at three timepoints (days 1, 7 and 14). However, capturing improvement was challenging for more severe lesions which take longer to have the improvement reflected in the UD score. For example, animals with severe UD lesions were subjectively improved at some time points, however the correlating score of the lesion did not change because the lesion length and character did not change enough to move to a lower score. Also, while one portion of the UD scoring system takes into account what region of the animal is affected to determine the overall score, we did not evaluate whether lesion location affected response to treatment. The same is true for lesion size. Future investigations may consider monitoring lesion improvement for a longer time period and evaluating whether treatment success was different based on lesion location or overall size. Similar to other portions of the UD score system used, itch was incorporated into the overall score but was not recorded separately. Fukayuma et al. found decreased scratching bouts following application of topical oclacitinib when mice were observed for an hour, however the mice in our study were only monitored for 2 minutes [19]. Evaluating scratching behavior alone following application of topical oclacitinib may be worthwhile as this is the primary treatment goal of oral oclacitinib in other species. To the authors’ knowledge, no reported scoring system would be able to better represent objective UD scores.

Another way to monitor for lesion improvement is to assess how long it takes for scores to reach 0 (resolution). We assessed this within our groups and did not find a significant change in time to resolution, however the NMT group was faster to have at least 50% of the group reach a treatment score of zero than the OCL or NTO groups. Although comparison between studies is difficult due to many variables and differing recruitment criteria, this can offer a rough comparison of different UD treatment modalities. Notably, the time all groups in the current study took to have at least 50% resolution is faster than that reported by Alvarado et al. for nail trim only (17d) and observation only (36d) [3]. Adams et al. found treatment with nail trims followed by one application of topical bacitracin-neomycin-polymixin B with hydrocortisone, Tresaderm, or Vetericyn had similar results with 14d median time to cure for all groups [2]. Of these studies, our NMT treatment group was the fastest to resolve 50% of cases at only 7 days. The authors suspect the quick resolution was due to the multifactorial treatment approach, utilizing nail trimming to reduce scratching abrasions, meloxicam for inflammation and pain management, and antibiotic ointment for secondary infections. To our knowledge, this is the first time this treatment regimen has been investigated for ulcerative dermatitis.

This also has an important animal welfare implication as the pain and distress caused by self-inflicted injury of UD lesions deserves consideration. This aspect of UD resolution in response to treatment with oclacitinib may warrant further study as a quicker resolution of clinical signs and ulcerative lesions is obviously beneficial to animal wellbeing. We have a moral obligation to relieve pain and distress in laboratory animals unless an institutional animal care and use committee approves otherwise for scientific reasons. Therapeutics offering faster healing or reduced discomfort from active lesions are worth pursuing as a refinement to the care of mice with ulcerative dermatitis. While difficult to directly compare results of this study to prior investigations, our treatment success was between 50–71% dependent upon the group. While this was lower than the 93% resolution reported in one study, it was higher than many of the other topical or novel treatment success rates in other reported studies [2]. However, it is important to recognize that we consider treatment success a UD score of 0 within 2 weeks of initiating treatment, whereas other investigations may have had different criteria for treatment to be considered successful and also followed clinical cases for resolution past the 2-week timeframe that we evaluated.

As more UD treatment options arise, it is important to consider the moral and scientific merit of maintaining and treating animals with UD. JAK inhibitors serve as a valuable option in the treatment of UD when other treatment modalities may not be a viable study option. In instances where non-steroidal anti-inflammatory drugs (NSAIDs) may alter immunological responses to treated animals or in instances where antibiotic therapy, either topical or systemic, might alter microbiomes of treated animals, JAK inhibitors may serve as a viable alternative. While oclacitinib has been shown to affect some inflammatory cytokines, the degree to which this may be relevant to specific studies is not yet fully understood [19]. However, the relatively narrow scope of cytokine inhibition by JAK inhibitors such as oclacinitib may make them a more viable option than NSAIDs or other treatments that have more wide-spread anti-inflammatory responses [15].

In conclusion, the results of this study show that topical oclacitinib used in the current capacity did not provide any additional benefit to resolve UD lesions. Although unsuccessful, this study demonstrates the feasibility of using JAK inhibitors in mice and we hope that further investigations to optimize the use of oclacitinib as a therapy for murine dermatitis are explored.

## Supporting information

S1 Data (PDF)

## Abbreviations

DMSO: dimethyl sulfoxide
JAK: janus kinase
NMT: nail trim plus meloxicam and topical triple antibiotic ointment group
NSAID: non-steroidal anti-inflammatory drug
NTO: nail trim only group
OCL: nail trim plus topical oclaticinib group
UD: ulcerative dermatitis
