# Supplementary material for: Evaluation of topical oclacitinib and nail trimming as a treatment for murine ulcerative dermatitis in laboratory mice
Source: PLoS One. 2022 Oct 18;17(10):e0276333. doi: 10.1371/journal.pone.0276333 (PMC9578627; doi:10.1371/journal.pone.0276333)
Supplement: S1 Data — (PDF) [file pone.0276333.s001.pdf]

MouseID	Tx	Score1	Score2	Score3	Sex	DOB	DateEnrolled	AgeAtTx	Strain
9	Apoquel	8	3	3	F	9/20/2019	9/11/2020	357	KVB/WT
10	Apoquel	9	6	6	F	5/7/2019	9/17/2020	499	TGAA
11	Apoquel	8	4	5	F	4/6/2020	10/2/2020	179	CCR2 Het
12	Apoquel	3	0	0	F	4/6/2020	10/2/2020	179	CCR2 Het
13	Apoquel	4	0	0	F	4/6/2020	10/2/2020	179	CCR2 Het
14	Apoquel	9	8	8	M	9/22/2019	10/5/2020	379	Ephx2-/-, SE H KO
21	Apoquel	7	0	0	F		11/11/2020		unknown
22	Apoquel	7	7	9	F		11/11/2020		unknown
23	Apoquel	7	0	0	F		11/11/2020		unknown
24	Apoquel	3	0	0	F		11/13/2020		2613MD, C57
25	Apoquel	8	5	5	M	2/22/2019	11/13/2020	630	Chop APOE
26	Apoquel	4	3	0	M	1/31/2019	11/13/2020	652	Mir21K
29	Apoquel	7	0	0	F		11/20/2020		unknown
35	Apoquel	8	7	5	F	7/1/2020	11/24/2020	146	NK30
2	Normal	5	0	0	F	9/29/2019	9/2/2020	339	C57
5	Normal	6	0	0	F	6/12/2020	9/4/2020	84	LC3
17	Normal	5	0	0	M	7/21/2019	10/20/2020	457	RLIPKO
18	Normal	6	0	0	M	3/6/2020	10/20/2020	228	KRAS LAT Het
19	Normal	3	0	0	M	2/20/2020	11/4/2020	258	Hal2flox
31	Normal	6	0	0	F	10/29/2019	11/24/2020	392	C57BL6
32	Normal	8	0	0	F	11/16/2018	11/24/2020	739	C57BL6
33	Normal	7	4	4	F	11/16/2018	11/24/2020	739	C57BL6
37	Normal	8	6	6	F	12/30/2019	12/21/2020	357	unknown
39	Normal	6	0	0	F		1/7/2021		unknown
40	Normal	3	0	0	F		1/13/2021		Cre ATF3
43	Normal	5	4	3	M	8/14/2020	1/15/2021	154	Cng Bmal
44	Normal	6	3	0	F	8/9/2019	1/15/2021	525	unknown
45	Normal	6	7	6	F	4/28/2020	1/18/2021	265	CCR2 Het
3	NT	4	0	0	M	2/2/2020	9/3/2020	214	transgenic
7	NT	7	0	0	F	6/12/2020	9/4/2020	84	LC3
8	NT	8	0	0	F	6/12/2020	9/4/2020	84	LC3
15	NT	6	8	7	F	6/16/2020	10/7/2020	113	Math 5
16	NT	7	0	0	F	12/3/2019	10/7/2020	309	unknown
20	NT	6	0	0	M		11/7/2020		unknown
27	NT	8	9	6	F		11/19/2020		C57BL6
28	NT	9	3	0	F		11/19/2020		C57BL6
30	NT	6	6	6	F		11/24/2020		Kras 0/0 (WT)
34	NT	8	4	0	M	7/16/2020	11/24/2020	131	WT
36	NT	7	0	0	F		12/4/2020		unknown
38	NT	6	2	0	F		12/21/2020		Kras LSL
41	NT	5	3	0	F		1/14/2021		unknown
4	NT	7	6	6	M	4/10/2020	9/3/2020	146	B6.129CCR2KO X C57 WT
